# RAGE in Cancer Lung: the End of a Long and Winding Road is in Sight

**DOI:** 10.3779/j.issn.1009-3419.2018.09.01

**Published:** 2018-09-20

**Authors:** Armando ROJAS, Ileana GONZÁLEZ, Paulina ARAYA

**Affiliations:** Biomedical Research Laboratories, Medicine Faculty, Catholic University of Maule, Talca, Chile

**Keywords:** Receptor of advanced glycation end-products, Cancer, Lung neoplasms, Lung physiology

## Introduction

The receptor of advanced glycation end-products (RAGE), is a type Ⅰ single-pass transmembrane protein, member of the multi-ligand immunoglobulin superfamily molecule, which was discovered in 1992, on account of its ability to bind the advanced glycation end-products (AGEs)^[1]^.

The mature RAGE protein is 404 amino-acids long and it is comprised by three domains; an extracellular domain, followed by a single hydrophobic transmembrane spanning region and a short cytosolic domain. From the structural point of view, the extracellular moiety comprises three Ig-like domains. The N-terminal domain has been assigned to the V-set of the Ig-like molecules, thereby referred as the V domain of RAGE. The two additional extracellular domains are known as C1 and C2. The VC1 tandem domain forms a functional unit characterized by the presence of a large positively charged patch and the C2 domain is negatively charged^[2]^.

In addition to the full-length receptor, RAGE undergoes extensive alternative splicing to produce a variety of transcripts with diverse functions, including a soluble antagonist variant, also known as endogenous secretory (esRAGE) as well as a truncated circulating soluble form (sRAGE) arising from the ectodomain of full-length RAGE, by the action of membrane associated-proteases, including the sheddase ADAM-10 and the matrix metalloproteinase-9 (MMP-9)^[3]^, as well as by G-protein coupled receptor mediated shedding^[4]^.

Of note, both soluble RAGE variants may function as a decoy for ligands, and thus preventing the interaction with the membrane anchored full-length RAGE^[5]^.

Although initially described as a receptor for advanced glycation end-products (AGEs), RAGE is now considered as a very promiscuous receptor displaying a very wide and diverse repertoire of ligands, and being able to bind some members of the S100/calgranulin family, such as S100A12, S100B, S100P, S100A4, S100A8/A9); HMGB1; adhesion molecules such as Mac-1, ameloid betapeptide, and many other damage signals^[6]^, and as such it has been classified as a pattern-recognition receptor^[7]^.

Many of RAGE ligands are not under the control of degradation mechanisms, in such a way, that they persist and accumulate at inflammation sites. Furthermore, ligation of RAGE not only causes an inflammatory gene expression profile but also a positive feed-forward loop, in which inflammatory stimuli activate nuclear factor-kappa B (NF-κB), which induces RAGE expression, followed by a sustained NF-κB activation, and thus perpetuating a continuous amplification loop^[8, 9]^.

One of the most intriguingly aspect of RAGE biology is related with the fact that in the majority of healthy adult tissues, RAGE is expressed at a low basal level and increased RAGE expression has been strictly associated with a diverse range of pathological events. However, pulmonary tissues express remarkably high basal levels of RAGE suggesting that RAGE may have a number of functions in the lung distinct from that which it holds in other tissues.

At present, several clinical studies have demonstrated a strong association of RAGE expression with the malignant potentialof various cancer types such as gastric cancer, colon cancer, common bile duct cancer, pancreatic cancer, prostate cancer, oral squamous cell carcinoma, and breast cancer among others^[10]^.

Although RAGE is highly expressed in normal alveolar epithelium, it is surprisingly and markedly reduced in lung carcinomas, RAGE over-expression in normal lung alveolar type Ⅰ epithelial cells is followed by rapid down-regulation upon malignant transformation, being associated with increased aggressiveness in non-small cell carcinomas and adenocarcinomas^[11-13]^.

During the last years, new reports have shed light on the role of RAGE in lungs. RAGE is highly expressed in the alveolar epithelial type 1 cells^[14]^.

These cells cover up to the 95%-98% of the alveolar surface area and thus forming a crucial barrier between the air filled alveolus and the blood capillary network. Of note, RAGE is closely related to the maintenance of normal morphological structure and function of the alveoli, ensuring alveolar stability. Strikingly, in this cell type, RAGE actively mediates the adhesion of cells to extracellular matrix proteins, as well as the interaction between two adjacent cells, and thus supporting the characteristic morphology of these cells by enhancing their spreading and flattening^[15, 16]^.

RAGE loss or diminished expression might contribute to the loss of polarization and differentiation. Therefore, the reduced expression levels of full-length RAGE may then result in an impaired functioning of lung epithelium by disrupting cell homeostasis and increase migratory and invasive behavior as well as concomitant oncogenic transformation^[17]^.

Redox regulation is crucial for cell signaling, and a disruption in redox regulation may then result in oxidative stress, which is a significant contributor to lung pathologies, including lung cancer, ROS can affect not only the phenotypic behavior of cancer cells but also their responsiveness to therapeutic interventions^[18-20]^.

As recently reported, RAGE is a contributor to redox regulation in alveolar epithelial cells, where RAGE knockdown markedly decreasekey regulators of redox signaling such as thioredoxin-1 superoxide dismutase 1, thioredoxin-like protein 1, and thioredoxin domain containing protein 17; and thus suggesting that RAGE is an active contributor to redox regulation^[21]^.

Of note, advancing stage of lung cancer display increased levels of oxidative stress, while levels of antioxidant molecules are markedly decreased^[22-24]^.

Strikingly, a very recent report combined two-stage functional annotation study based on two data bases (TCGA (http://cancergenome.nih.gov) and GEO (http://www.ncbi.nlm.nih.gov/geo/), as well as *in vitro* transfection experiments on lung cancer cells has provided strong evidence that RAGE may act as a tumour suppressor gene to regulate lung cancer development^[25]^.

In summary, and after more than one decade of intense efforts to understand the biology of RAGE in lungs, as well as to unravel the very particular role of receptor in lung cancer, the end of the road is at sight.
